# Publisher Correction to: Acta Neuropathologica Communications, volume 7

**DOI:** 10.1186/s40478-019-0784-5

**Published:** 2019-08-14

**Authors:** Acta Neuropathologica Communications

**Affiliations:** London, UK

**Publisher Correction to: Acta Neuropathologica Communications, volume 7**

An error occurred during the publication of a number of articles in *Acta Neuropathologica Communications*. Several articles were published in volume 7 with a duplicate citation number.

In this correction article the old and new citation metadata are published in Table 1.

**Table 1:** Overview of incorrect and correct citation metadata

DOI	Incorrect citation number	Correct citation number
10.1186/s40478-019-0734-2	1	85
10.1186/s40478-019-0736-0	4	84
10.1186/s40478-019-0737-z	14	96
10.1186/s40478-019-0738-y	10	87
10.1186/s40478-019-0739-x	2	95
10.1186/s40478-019-0740-4	13	86
10.1186/s40478-019-0741-3	6	93
10.1186/s40478-019-0742-2	12	90
10.1186/s40478-019-0743-1	9	91
10.1186/s40478-019-0744-0	5	88
10.1186/s40478-019-0745-z	7	89
10.1186/s40478-019-0746-y	8	92
10.1186/s40478-019-0747-x	11	94
10.1186/s40478-019-0748-9	3	98
10.1186/s40478-019-0749-8	15	99
10.1186/s40478-019-0751-1	16	97

The original articles have been updated. The publisher apologizes for the inconvenience caused to our authors and readers.

